# Strawberry Production in Soilless Culture Systems: A Comparative Analysis of Volatile Metabolites, Quality, and Sensory Traits in Three Cultivars

**DOI:** 10.3390/foods15061072

**Published:** 2026-03-18

**Authors:** Livia Malorni, Tiziana Di Renzo, Cristina Matarazzo, Milena Petriccione, Elvira Ferrara, Giuseppe Capriolo, Gianluca Baruzzi, Paolo Sbrighi, Rosaria Cozzolino

**Affiliations:** 1 Institute of Food Science, National Council of Research (ISA-CNR), Via Roma 64, 83100 Avellino, Italy; livia.malorni@isa.cnr.it (L.M.); tiziana.direnzo@isa.cnr.it (T.D.R.); 2 Department of Agriculture, Environmental and Food Sciences, University of Molise, 86100 Campobasso, Italy; c.matarazzo3@studenti.unimol.it; 3 Council for Agricultural Research and Economics (CREA), Research Centre for Olive, Fruit and Citrus Crops, Via Torrino 2, 81100 Caserta, Italy; milena.petriccione@crea.gov.it (M.P.); elivira.ferrara@crea.gov.it (E.F.); giuseppe.capriolo@crea.gov.it (G.C.); 4 Council for Agricultural Research and Economics (CREA), Research Centre for Olive, Fruit and Citrus Crops, Via La Canapona, 1 Bis, 47121 Forlì, Italy; gianluca.barbuzi@crea.gov.it (G.B.); paolo.sbrighi@crea.gov.it (P.S.)

**Keywords:** soilless cultivation system, physico-chemical traits, microbiological quality, volatile organic compounds, sensory analysis

## Abstract

Strawberry aroma and flavor are key determinants of consumer acceptance and market value, yet their relationship with physico-chemical and functional traits remains complex and cultivar-dependent. This study aimed to characterize the volatile profile, quality parameters, antioxidant capacity, microbial load, and sensory attributes of three strawberry cultivars (‘Rossetta’, ‘Melissa’, and ‘Gioelita’) grown in soilless culture systems and harvested at the commercial ripening stage. ‘Melissa’ showed significantly higher total soluble solids (8.65 °Brix) than ‘Rossetta’ (7.78 °Brix) and ‘Gioelita’ (7.47 °Brix), while titratable acidity was highest in ‘Gioelita’ (4.97 mg CA/L). Regarding phytochemical traits, ‘Melissa’ exhibited the greatest total polyphenol, flavonoid, and antioxidant capacity values, followed by ‘Rossetta’ and ‘Gioelita’. Sixty-four volatile organic compounds (VOCs) were identified, semi-quantified, and combined with physico-chemical and sensory data related to odor and taste perception. Principal component analysis was applied to evaluate cultivar discrimination and identify the key discriminatory volatiles. The results revealed clear separation among cultivars based on their compositional and sensory profiles. ‘Rossetta’ was characterized by a higher abundance of esters, lactones, and mesifuran and received the highest sensory scores for sweetness and overall flavor, consistent with its elevated anthocyanin content. ‘Gioelita’ was associated with key esters contributing to strawberry flavor and with higher titratable acidity and perceived acidity. ‘Melissa’ showed a balanced volatile composition, higher antioxidant capacity, and greater phenolic content but also had higher microbial counts. Overall, the integration of chemical and sensory analyses provided useful insights into cultivar-specific quality traits relevant for breeding and production strategies.

## 1. Introduction

Strawberries (*Fragaria* × *ananassa* Duch.), called ‘false fruit’, are highly valued worldwide for their vibrant red color and appealing sensory traits related to texture, aroma, and taste [1]. This fruit has been qualified as a bio-functional food as it constitutes a rich natural source of bioactive molecules, including phenolic acids and vitamins, which provide a range of health benefits [2].

Among berry plants, strawberries are the most widely demanded, with 95.6 × 10^3^ tons produced globally in 2022, due to consumers’ awareness of their positive effects on health [2].

Commercial strawberry production has traditionally relied on soil-based cultivation, with plant propagation mainly occurring through stolons [3]. Nevertheless, conventional soil systems can be affected by several constraints, including low soil fertility, water scarcity, limited land availability, and substantial yield losses due to soil-borne pathogens and nematodes [4,5].

Recently, traditional soil cultivation methods have been replaced by innovative techniques, such as soilless substrate-based greenhouse production systems, arising as a new trend in strawberry production [6]. These systems are already widely adopted in strawberry production, as they allow precise control of mineral nutrition through the targeted management of nutrient solutions, thereby improving nutrient use efficiency [7]. Several studies have demonstrated that soilless cultivation enhances plant growth, physiological performance, fruit yield, and quality in strawberries, while also ensuring higher sanitary standards compared to soil-based systems [8,9,10]. Overall, soilless farming systems represent a sustainable and efficient strategy for overcoming soil-related constraints using new growing media in each production cycle and extending the production season under controlled environmental conditions [7].

These new methods are very attractive from both environmental (decreased use of water and pesticides) and producers’ perspectives (increased yields, longer plant survival, reduced use of water) [6]. Regarding the nutritional aspects, evidence is inconsistent as some studies indicate improvements in nutritional quality under soilless cultivation [6], while others report no statistically significant variation or higher performances of soil-grown produce in selected nutritional parameters [11].

With the expansion of the strawberry market and increasing consumers’ expectations concerning fruit quality, research attention has moved beyond traditional parameters such as yield and general fruit quality to include aroma characteristics [6]. Strawberry flavor is a crucial parameter that drives consumer acceptance and market value. It is formed via several metabolic pathways, resulting in a complex mixture of volatile organic compounds (VOCs) [12,13]. So far, in strawberries, more than 360 aroma metabolites, principally belonging to esters, terpenes, and furanones, have been identified [14]. All these compounds, through content gradients and synergistic effects, participate in shaping the strawberry’s unique sensory traits, namely fruity, floral and creamy [15].

Previous studies investigating the distinctive VOC profiles of commercial strawberry cultivars grown in soilless cultivation systems using headspace solid-phase microextraction gas chromatography–mass spectrometry (HS-SPME-GC-MS) revealed that some cultivars exhibit a wider range and higher amounts of aromatic compounds [6,16,17]. Since strawberry sensory attributes and nutritional traits are largely influenced by genetic factors intrinsic to each cultivar, these findings indicate that the aroma of strawberries produced in soilless systems is primarily determined by the cultivar itself, thereby influencing consumer purchasing decisions [6,18]. Therefore, to provide basic information for variety breeding and harvest optimization, the investigation of the volatile profile and the sensory traits of different strawberry varieties grown in such innovative systems is essential.

This study represents the first comprehensive evaluation of the qualitative and sensory performance of three commercial strawberry cultivars (‘Melissa’, ‘Gioelita’, and ‘Rossetta’), adapted to warm Mediterranean-type climates and cultivated under a soilless production system.

Fruits were harvested at the same stage of ripeness, and analyses were conducted to assess their physical and chemical traits, detailed volatile organic compounds (VOCs) profiles, microbiological status, and sensory attributes.

## 2. Materials and Methods

### 2.1. Samples and Experimental Design

The experimental field belonging to Cooperativa Sole, a cooperative specializing in strawberry production and known for its commitment to natural farming methods and eco-compatible techniques, is located in Parete (Caserta, Italy). The cultivation of three strawberry cultivars (‘Melissa’, ‘Gioelita’, and ‘Rossetta’) with a plant lifespan of about 9 months was characterized by a high plant density (150,000 p/ha), and the cultivars were planted on a structure raised above the ground consisting of commercial substrate (Grotec^®^ 21, Agrochimica, Bolzano, Italy). Irrigation is fully managed by the fertigation unit, with two irrigation events per day, two minutes per sector. Each sector covers approximately 320 m^2^, with 7 raised beds per greenhouse. Each raised bed receives about 30 L per irrigation event, totaling approximately 60 L per day per bed. The EC and the pH are set between 1.0 and 1.5 ms cm^−1^ and between 5.0 and 5.6, respectively. The nutrient solutions were composed of calcium nitrate, ammonium nitrate, potassium nitrate, magnesium sulfate, and micronutrients (boron, copper, iron, manganese, molybdenum, and zinc).

Temperature and relative humidity inside the greenhouse were regulated through natural ventilation, with the frontal vents opened daily to adjust internal conditions according to the prevailing environmental conditions.

For each cultivar, the field experiment was arranged in three blocks, with twenty plants per block located on different rows. Fruits were collected at the commercial ripening stage in May (20th and 27th, 2025) from each block and pooled within the block; therefore, each block represented one independent biological replicate. Strawberry fruits were harvested at the commercial ripening stage in May (20th and 27th, 2025), transported to the CREA–Research Centre for Olive, Fruit and Citrus Crops laboratory, and selected for uniformity in size, color, and shape for further analysis.

### 2.2. Reagents and Chemicals

Gallic acid (≥98.0%), catechin (≥99.0%), 2,2′-Azino-bis (3-ethylbenzothiazoline-6-sulfonic acid) diammonium salt ABTS (≥98%), Cyanidin 3-glucoside chloride (≥98%), and Trolox (≥98%) were obtained from Merck (Darmstadt, Germany). All reagents were of analytical grade and used without further purification.

### 2.3. Strawberry Fruit Quality

#### 2.3.1. Physio-Chemical Parameters

Firmness was assessed on two opposite sides of 15 fruits per cultivar using a digital penetrometer (Turoni, Forlì, Italy), and the results were expressed in kg/cm^2^. Total soluble solids (TSS) content was determined on juice obtained from ten fruits per cultivar using a digital refractometer (DBR35, Sinergica Soluzioni, Pescara, Italy) and expressed in °Brix. Titratable acidity (TA) was quantified by acid-base titration with 0.1 N sodium hydroxide (NaOH) to an endpoint of pH 8.1, and results were expressed as grams of citric acid per liter of juice (g CA/L). Juice pH was measured using a digital pH meter (XS PH 50 VIO LAB, model 2021, Turin, Italy). The color of the fruit skin was evaluated on two opposite sides of 15 fruits per cultivar using a Minolta CR5 colorimeter (Minolta Camera Co., Tokyo, Japan). The color parameters were acquired according to the CIELAB color system, recording the values of L* (lightness), a* (color axis from green to red), and b* (color axis from blue to yellow). The chroma and hue angle (H*) were calculated according to the method described by McGuire [19].

#### 2.3.2. Bioactive Compounds

Bioactive compounds were extracted by homogenizing strawberry tissue in an 80% methanol solution (*v*/*v*) (1:5). Total polyphenol content (TP) was determined using the Folin–Ciocalteu method [20]. The absorbance of the reaction mixture, containing 20 µL of methanolic extract, was measured at 765 nm, and the results were expressed as mg of gallic acid equivalents (GAE) per 100 g of fresh weight (FW).

Total flavonoid content (TF) was determined according to the method of Zhishen et al. [21]. The absorbance of the assay mixture containing 200 µL of methanolic extract was measured at 510 nm, and the results were expressed as mg of catechin equivalents (CE) per 100 g of FW.

The determination of total anthocyanins (ANT) was performed using the differential pH spectrophotometric method [22]. The extracts (100 µL) were incubated in buffers at pH 1.0 and 4.5, and the absorbance was measured at 510 and 700 nm; the monomeric anthocyanin content was expressed as mg of cyanidin-3-glucoside equivalents (C3GE) per 100 g of FW.

Total antioxidant capacity (AC) was evaluated using the 2,2′-azino-bis (3-ethylbenzothiazoline-6-sulfonic acid) (ABTS) assay, which determines the ability of the extracts to reduce the cationic radical ABTS•^+^, resulting in a decrease in absorbance at 720 nm [23]. Antioxidant capacity was expressed as Trolox equivalents (TE) per g of FW.

### 2.4. Analysis of VOC Profiles

Volatile compound profiles in strawberry samples were obtained by headspace solid-phase microextraction (HS-SPME) coupled with gas chromatography/mass spectrometry (GC/MS), following the method described by Cozzolino et al. [24].

For the HS-SPME extraction, 1 g of each cultivar was weighed in a 20 mL screw-on cap HS vial, and 0.3 g of NaCl and 1.5 μL of 3-octanol (20 ppm), used as the internal standard (IS), were added. To obtain a representative sample from each harvest date, 1 Kg of fresh fruit was homogenized, and 1 g from the whole sample was collected. Vials were sealed with a Teflon septum and an aluminum cap (Chromacol, Hertfordshire, UK) and incubated at 40 °C for 10 min with constant stirring (250 rpm). Afterwards, VOCs were allowed to be adsorbed onto a DVB/CAR/PDMS (50/30 µm) fiber surface by keeping the fiber in the vial for 20 min. All steps were performed automatically using an autosampler MPS 2 (Gerstel, Mülheim, Germany).

Prior to the first use, fibers were conditioned as indicated by the manufacturer, but below the maximum suggested temperature. Prior to the initial daily analysis, the fiber was conditioned for 5 min at the operating temperature of the injector port of the GC.

VOC detection was achieved using a gas chromatograph (GC 7890A) combined with a mass spectrometer (5975 C, Agilent Technologies, Santa Clara, CA, USA). The HS-SPME fiber was automatically inserted into the injector port of the GC for 10 min at 240 °C to allow the direct desorption of VOCs to a capillary column HP-Innowax. Oven temperature was initially maintained at 50 °C for 3 min, then increased to 160 °C at 5 °C min^−1^, kept at that temperature for 1 min, ramped to 250 °C at 10 °C min^−1^, and maintained for 2 min. Mass spectrometry analysis was achieved at an ionization energy of 70 eV with the detector operating in a mass range between 30 and 300 u and a scanning speed of 2.7 scans/s. VOCs were identified by mass spectra based on the NIST05/Wiley07 libraries by matching the linear retention indices (LRIs) with the literature values and by using commercial standards, if available. For each strawberry cultivar at each harvest date, VOC analyses were conducted in three technical replicates following a randomized sequence in which blanks (analyses of coating fibers without any extraction procedure) were also run. Semi-quantitative data of each volatile were expressed as relative peak area (%RPA) obtained by the ratio between the peak area of the metabolite and that of the IS multiplied by the known concentration of IS added to the sample. The results are reported as micrograms per kilogram (μg/kg) of IS equivalents. Areas were measured from the total ion chromatogram (TIC).

### 2.5. Microbiological Analysis

Samples of strawberries from each cultivar were analyzed to verify their microbiological quality, as reported by Reale et al. [25]. Briefly, 10 g of each sample was aseptically placed into a sterile stomacher bag and diluted with 90 mL of sterile physiological solution (9 g/L NaCl). After homogenization for 1 min using a Stomacher apparatus (Bag-Mixer 400, Interscience, Saint-Nom-la-Bretèche, France), the samples were serially diluted and plated. Microbial groups were enumerated as follows: total mesophilic bacteria on Plate Count Agar after 48 h incubation at 28 °C; Enterobacteriaceae on VRBGA after 36 h incubation at 37 °C; total and fecal coliforms on VRBA after 36 h at 37 °C and 44 °C, respectively; and yeasts and molds on Potato Dextrose Agar (pH 3.5) after 5–7 days at 28 °C. Incubation was performed using FALC incubators (Treviglio, Italy).

*Salmonella* spp. detection was carried out following the procedures described in ISO 6579, as reported by Di Renzo et al. [26]. For the enumeration of *Escherichia coli*, a selective chromogenic medium (TBX, Tryptone Bile Glucuronic Agar, OXOID (Milan, Italy)) was used, and the plates were incubated at 44 °C for 18–24 h (FALC instruments S.R.L., Treviglio, Italy) as indicated in ISO 16649 and reported by Ortiz-Solà et al. [27]. Viable counts were expressed as the logarithm of colony-forming units per gram of fresh fruit (Log CFU/g). All culture media were obtained from OXOID (Milan, Italy), and each microbiological analysis was carried out in triplicate.

### 2.6. Sensory Analysis

Sensory evaluation was conducted by a panel of sixteen trained assessors (both sexes, aged 40–60 years). Prior to the analysis, panelists underwent a three-week training period consisting of 1 h sessions aimed at familiarizing them with strawberry sensory attributes. During these sessions, descriptors and definitions related to odor, texture, and taste were developed through brainstorming and panel consensus. Assessors were provided with commercial strawberry samples at different ripening stages, as well as basic taste standard solutions (Table S1), to establish reference anchors for the 9-point intensity scale [28].

The panel assessed odor traits, including sweet, herbaceous (green), typical strawberry odor, overripe/fermented and pungent odor intensity and taste attributes, including sweetness, sourness, strawberry taste intensity and overall taste. Additionally, texture was described and analyzed in terms of firmness, juiciness, seediness and the crunchiness of the fruit [29].

Sensory analysis was performed in three-day sessions for each strawberry cultivar harvested at both harvest dates (20 and 27 May 2025). On the day of analysis, the samples were rinsed, and two fruits for each cultivar were placed in a plastic dish, coded with a three-digit number and served at room temperature. The sensory evaluation area was equipped with eight booths, air-conditioned at 20 ± 2 °C and with 50 ± 5% relative humidity, and lit with a white light at 850 Lux.

The order of presentation was completely balanced pairwise, and data were acquired by a printed form. Natural water was provided to cleanse the palate between samples.

### 2.7. Statistical Analysis

One-way analysis of variance (ANOVA) was performed to highlight differences among physio-chemical parameters, bioactive compounds and VOC profiles in the three strawberry cultivars. Significant differences were detected at *p* < 0.05 by Tukey’s post hoc test. The collected sensory data were submitted to a two-way ANOVA (main factors: product and assessors) with interactions, significance level fixed at *p* ≤ 0.05, using Panel Check v.1.4.2 (Nofima, Tromso, Norway). The differences in sensory attributes between samples were assessed by applying the Tukey test for multiple comparisons of means, taking into account the *p*-values 0.05, 0.01, and 0.001 for significance levels. Physico-chemical analyses were carried out in duplicate. Mean values and standard deviations for the two harvests were analyzed using one-way ANOVA, followed by Duncan’s post hoc test (*p* < 0.05). The microbiological count data were analyzed using GraphPad Prism version 9.0 (GraphPad Software Inc., San Diego, CA, USA). Results are expressed as mean values ± standard deviation (SD). Statistical differences among groups were assessed by two-way analysis of variance (ANOVA), followed by Tukey’s multiple-comparison test. A confidence level of 95% was applied, and differences were considered statistically significant at *p* ≤ 0.05.

Principal component analysis (PCA) was performed using the correlation matrix under the extraction settings, thereby standardizing variables through mean-centering and scaling to unit variance (z-score transformation) among the quality parameters, the sensory traits and the VOCs data to detect the principal components responsible for the majority of the variations within the dataset.

Correlations among all parameters in the dataset were analyzed using Pearson’s correlations (*p* < 0.05 and *p* < 0.01). All the analyses were executed using SPSS Version 20.0 (SPSS Inc., Chicago, IL, USA).

## 3. Results and Discussion

### 3.1. Physico-Chemical and Qualitative Traits in ‘Melissa’, ‘Gioelita’ and ‘Rossetta’ Strawberry Cultivars

Significant differences among cultivars were observed for several physico-chemical and qualitative traits (Table 1). Physico-chemical traits influence consumer acceptance and shelf life [30,31], as they predominantly reflect the concentrations of glucose, fructose, and sucrose. Soluble sugars play a central role in determining fruit sweetness and, consequently, consumer acceptance [32]. TSS was significantly higher in ‘Melissa’ (8.65 °Brix) compared to ‘Rossetta’ (7.78 °Brix) and ‘Gioelita’ (7.47 °Brix). Despite being present at lower concentrations than sugars, organic acids in strawberries play a critical role in shaping the overall flavor, contributing to its complexity [33]. Titratable acidity (TA) was highest in ‘Gioelita’ (4.97 mg CA/L), while ‘Rossetta’ and ‘Melissa’ did not differ significantly. Consequently, pH values were lowest in ‘Gioelita’ and highest in ‘Rossetta’, with ‘Melissa’ showing intermediate values (Table 1).

Fruit softening is a complex physiological process involving the coordinated action of multiple cell wall-modifying enzymes and structural proteins. Changes in cell wall metabolism over time, together with the regulation of related gene expression, have been widely documented in several fruit species, including strawberries [34].

Color is largely determined by the anthocyanin content, which varies among cultivars and influences the visual perception of ripeness and quality. In the present study, no significant differences were detected among cultivars for L*, chroma (C), and hue angle (H°), indicating comparable basic color characteristics despite the differences in anthocyanin concentration. These results suggest that, while genotypes may differ in anthocyanin composition, the overall color intensity remains comparable, as suggested by Virgen-Ortiz et al. [35]. Regarding phytochemical traits, ‘Melissa’ showed the highest levels of total polyphenols (TP), flavonoids (TF), and antioxidant capacity (AC), followed by ‘Rossetta’ and ‘Gioelita’, depending on the parameter considered. In contrast, anthocyanin content (ANT) was highest in ‘Rossetta’, intermediate in ‘Gioelita’, and lowest in ‘Melissa’ (Table 1). Several studies reported that different strawberry cultivars exhibited differences in bioactive compounds and antioxidant capacity, suggesting that each cultivar differs in its bioactive compound profiles and, consequently, in its potential nutritional contribution. Identifying and promoting varieties with higher phytochemical content could, therefore, be important for both future research, breeding and dietary recommendations [36,37].

### 3.2. Characterization of the Volatile Profile in ‘Melissa’, ’Gioelita’ and ‘Rossetta’ Strawberry Cultivars

A total of 64 VOCs, namely esters (21), aldehydes (9), alcohols (8), acids (8), terpenes (13), lactones (4), and furanones (1), were detected at the two different sampling times in the three strawberry cultivars, namely ‘Rossetta’, ‘Melissa’ and ‘Gioelita’. The detected VOCs are detailed in Table 2, which also includes the abbreviation code, the experimental and literature LRIs, the identification methods and the relative concentrations (μg/kg) for the compounds assigned. Most of them were previously observed in several strawberry cultivars [15,38,39].

Regarding method repeatability (RSD%), our results showed adequate values. Specifically, for the main key odorants, RSD% was 3.3% for methyl butanoate, 2.7% for methyl hexanoate, 4.5% for γ-decalactone and intermediate precision for benzenmethanol and γ-dodecalactone (8.7% and 8.5%, respectively). By using the signal-to-noise method, the limit of detection (LOD) and the limit of quantification (LOQ) for the same key volatiles were, respectively, 1.5 and 5.0 μg/kg for methyl butanoate, 3.6 and 12 μg/kg for methyl hexanoate, 0.3 and 1 μg/kg for benzenmethanol, 0.3 and 1.0 μg/kg for γ-decalactone, and 0.9 and 3.0 μg/kg for γ-dodecalactone.

One-way ANOVA performed on the semi-quantitative (RPA%) HS-SPME/GC-MS data (Table 2) revealed pronounced inter-cultivar differences in the volatilomic signatures, as reported in Supplementary Table S1, which shows that, except for four compounds, namely benzyl acetate (E21), 2-heptenal (Ald4), decanal (Ald7) and 1-octanol (Alc7), all VOCs were statistically significant (*p* < 0.05).

**Figure 1:**
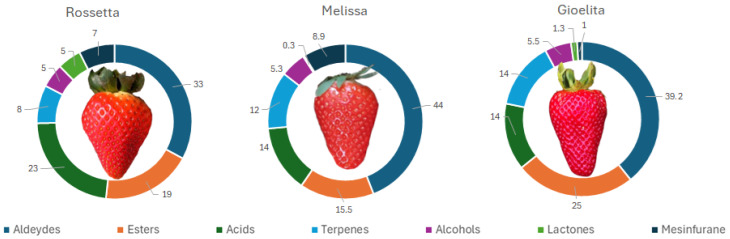
Contribution of each chemical class to the total volatile profile of investigated strawberry cultivars.

Figure 1 presents the contribution of each chemical class to the total volatilomic profile of each strawberry cultivar.

Aldehydes were the main chemical class in all three cultivars at both sampling points, accounting for about 33, 44 and 39% of all volatiles detected in ‘Rossetta’ (R), ‘Melissa’ (M) and ‘Gioelita’ (G), respectively (Table 2). Among aldehydes, 2-hexenal (Ald3) was the main component in all three samples (28, 41 and 36% in R, M and G, respectively), followed by hexanal (Ald1) (Table 2). According to the literature, C6 aldehydes (including hexanal and 2-hexenal), biosynthesized by the lipoxygenase (LOX) pathway, dominate the VOC profiles in the initial maturation steps, affecting the early-stage flavor of unripe strawberries with a typical green and herbaceous aroma [39].

Esters were the second most abundant chemical class of all VOCs in ‘Melissa’ (15.5%) and in ‘Gioelita’ (25%) (Table 2). The main components in all strawberries were methyl butyrate (E1) (5,5, 6 and 8.5% in R, M and G, respectively) and methyl hexanoate (E7) (5,5, 5 and 9% in R, M and G, respectively), followed by trans-2-hexen-1-ol acetate (E12), hexyl butyrate (E14) and trans-2-hexenyl butyrate (E16), albeit in lower relative amounts (Table 2).

Acids showed the highest amounts in ‘Rossetta’ (23%). They were the third most representative class in ‘Melissa’ (14%), while in ‘Gioelita’, terpenes and acids showed a similar content, both representing 14% of the total VOCs fraction. Among acids, hexanoic acid was the most representative compound in all cultivars (19.5, 11.5 and 11.5% compared to the total volatile content in R, M and G, respectively) (Table 2).

‘Gioelita’ seemed to be the richest in terpenes (14%), among which linalool (T6), the main compound in all cultivars (5, 7 and 10% of all volatiles detected in R, M and G, respectively) (Table 2), has been recognized to enhance the floral and citrus notes also in strawberry aroma [40].

Regarding alcohols, trans-2-hexen-1-ol (Alc5) (3, 2.6 and 3.7% of all volatiles detected in R, M and G, respectively) and 1-hexanol (Alc2), with green and fresh aroma, were the most abundant in all cultivars (Table 2).

Lactones and furanones have been demonstrated to be among the most significant VOCs in impacting the strawberry flavor [38]. Among the three cultivars, ‘Rossetta’ presented the highest concentration of lactones (5% of all VOCs), mainly γ-decalactone (4.6%), while in ‘Melissa’ and ‘Gioelita’, they contributed 0.3 and 1.3%, respectively, of the total volatile profile (Table 1). On the other hand, ‘Melissa’ (8.9%) and ‘Rossetta’ (7.5%) were the richest in mesifuran (F1), which only accounted for 1% of the total VOC content in ‘Gioelita’.

The detection of aldehydes as the main volatile class, together with the observation of a high number of esters, the presence of linalool (T6), of several lactones, of hexanoic acid (A5) and of mesifuran (F1) in all cultivars suggest that the three strawberry samples are characterized by a mid-maturation stage, as, with the exception of aldehydes, most of these compounds have been reported to increase from the middle ripening step [39,41]. As demonstrated by previous studies, during ripening an increased lipid oxidation and hydrolysis are observed, which drive the transformation of aldehydes into esters and alcohols by the activation of both alcohol acyltransferase (AAT) and alcohol dehydrogenase (ADH) [42]. Consequently, across ripening, aldehydes decline, while terpenes (mainly linalool and cis-nerolidol), esters (methyl butyrate, methyl hexanoate, 2-hexen-1-ol acetate), lactones (γ-decalactone) and furanones (mesifuran) gradually increase, shaping the typical strawberry-like aroma [39]. Consequently, our results suggest that, even if strawberries were harvested at commercial ripening, the soilless cultivation method shaped their VOC profile through an enrichment in aldehydes.

It should be underlined that furaneol was absent in all cultivars, although this volatile is considered a key aroma compound in strawberry [38,43]. Studying the effect of harvest time on the volatiles of twenty-five strawberry genotypes has demonstrated that some VOCs, including furaneol, were mostly affected by the genotype rather than by the development stage. Conversely, several studies have reported that the content of mesifuran and furaneol considerably increased along the ripening of ‘Candonga’ strawberries, aligning with the fact that the enzymes involved in the formation of these molecules show the highest activity in the fruit at the full red stage [24,38,39,43,44]. To the authors’ knowledge, the characterization of the volatile profiles of the three investigated cultivars has not yet been reported in the literature; thus, it is difficult to explain if the absence of furaneol is due to genotype or to the particular soilless cultivation employed in this study.

Accumulating evidence shows that VOCs synergistically act to enhance the complexity and layering of strawberry scent [39]; thus, a sensory evaluation was performed on ‘Rossetta’, ‘Melissa’ and ‘Gioelita’.

### 3.3. Sensory Profiles of ‘Melissa’,’Gioelita’ and ‘Rossetta’ Strawberry Cultivars

**Figure 2:**
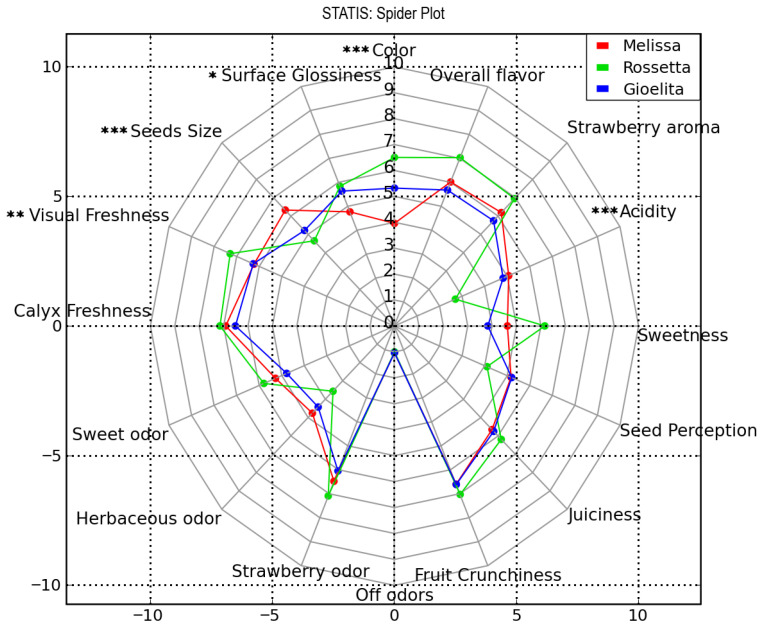
Attribute intensity defined by sensory panel for ‘Melissa’ (red), ‘Rossetta’ (green) and ‘Gioelita’ (blue). Attributes indicated by *, **, and *** have significant *p*-values (* 0.05, ** 0.01, and *** 0.001) by Tukey’s post hoc test.

The three strawberry varieties presented differed in their overall sensory profiles (Figure 2), which were built by using the data listed in Table S2. A spider plot, obtained using the STATIS analysis method in Panel Check, allowed the decomposition of the dataset variation into principal components and enabled the visualization of both assessor performance and sample average scores in the principal component space. Detailed results are listed in Table S2, while panel repeatability metrics are reported in Table S3. Specifically, ‘Melissa’ prevailed for *Seeds size* and *Strawberry odor* and *aroma*; ‘Rossetta’ recorded the highest intensity of *Color*, *Surface glossiness*, *Visual* and *Calyx Freshness*, and *Overall flavor*; while ‘Gioelita’ showed the highest value of *Acidity*. Conversely, ‘Melissa’ registered the lowest *Color* intensity and *Surface glossiness*, ‘Rossetta’ was the least acidic and recorded the lowest level of *Herbaceous odor*, smaller *Seeds size* and lower *perception*; while ‘Gioelita’ showed the lowest values of *Visual freshness*, *Calyx freshness*, *Sweet odor*, *Strawberry odor*, *Sweetness* and *Overall flavor*. All three varieties expressed similar levels of *Juiciness* (Figure 2).

### 3.4. Microbiological Quality of Strawberries

**Figure 3:**
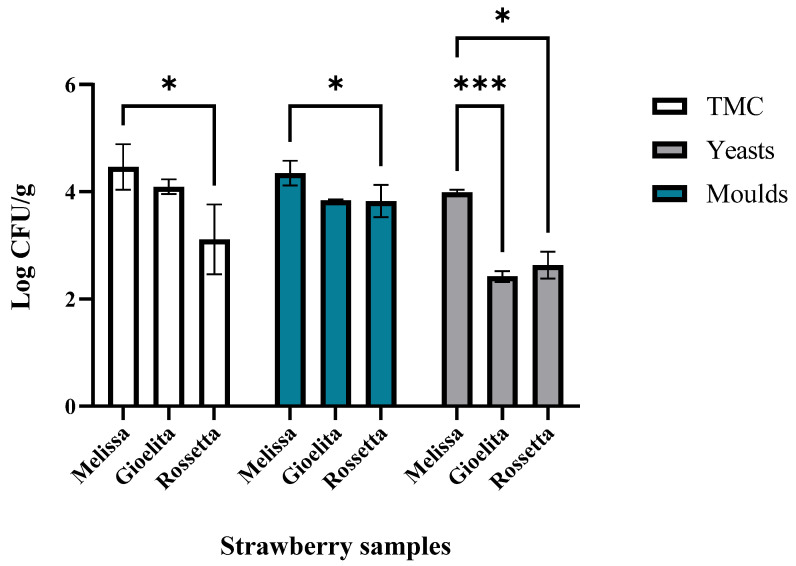
Microbial load (Log CFU/g) of TMC (total mesophilic count), molds, and yeasts in different cultivars of fresh strawberries. Significant *p*-value (*p* ≤ 0.05), (* 0.05 and *** 0.001).

Figure 3 presents the outcomes of the microbiological analyses performed on the strawberry cultivars.

The total aerobic mesophilic bacteria count (TMC) ranged from 3.11 ± 0.65 Log CFU g^−1^ (‘Rossetta’) to 4.46 ± 0.42 Log CFU g^−1^ (‘Melissa’). A statistically significant difference was found between samples from the ‘Melissa’ and ‘Rossetta’ cultivars (*p* = 0.0334).

These findings are in agreement with previous studies, which have reported that the total aerobic mesophilic count of fresh strawberries typically remains below 5 log CFU g^−1^, reflecting the generally low levels of microbial contamination associated with freshly harvested fruit and good agricultural and handling practices [27,45].

Microbiological contamination of strawberries can arise from multiple sources and at various points in the production chain, from harvesting to post-harvest handling and storage, leading to a swift decline in fruit quality. Their naturally short shelf life further complicates the marketing of fresh strawberries, as their high perishability makes them especially susceptible to fungal contamination [46].

Fungi levels in fresh strawberry samples were similar to those obtained in TMC. In detail, mold levels ranged between 3.83 ± 0.30 Log CFU g^−1^ (‘Rossetta’) and 4.33 ± 0.25 Log CFU g^−1^ (‘Melissa’). As in the case of TMC, a significant difference in mold load levels (Log CFU/g) between samples of the ‘Melissa’ and ‘Rossetta’ cultivars (*p* = 0.0107) was recorded.

The mold counts observed in our study were in line with those described in the literature. Previous authors have emphasized that deterioration due to mold proliferation is a significant constraint during the storage and commercialization of strawberries, restricting their refrigerated shelf life to approximately three days [47,48,49]. This limited storage window is mainly due to their high respiration rate, elevated moisture content, and continuous metabolic activity. Furthermore, their delicate structure and vulnerability to microbial decay greatly hinder effective storage and distribution. Yeast counts were lower than mold levels and ranged between 2.42 ± 0.10 Log CFU g^−1^ (‘Gioelita’) and 3.99 ± 0.05 Log CFU g^−1^ (‘Melissa’).

Fresh strawberries of the ‘Melissa’ cultivar exhibited significantly higher yeast levels than samples of the ‘Gioelita’ (*p* = 0.0002) and ‘Rossetta’ (*p* = 0.0281) cultivars.

Our findings align with those of other studies [50,51] reporting yeast populations of approximately 4 log CFU g^−1^ in fresh strawberries. Counts of Enterobacteriaceae, total coliforms, and fecal coliforms were all below 1 log CFU g^−1^. *Salmonella* spp. and *Escherichia coli* were not detected in any of the analyzed samples, consistent with previous reports [27]. Overall, the microbiological analyses of the fresh strawberry samples indicate that they were microbiologically safe, as all assessed parameters met the hygiene and safety criteria established by European Commission Regulation (EC) No. 2073/2005 [52].

### 3.5. PCA

Principal component analysis (PCA) was performed to explore potential correlations among the quality parameters, sensory attributes, and VOC profiles, with the aim of identifying the features that most effectively differentiate the three strawberry cultivars. For the PCA, the sensory attributes related to the olfactory and taste sensations (*Herbaceous odor*, *Sweet*, *Acidity*, *Sweet odor*, *Strawberry odor*, *Strawberry aroma*, *Overall flavor* and *Off odors*) were considered, as they strongly correlate among themselves. When odor and taste stimuli are assessed together, in fact, the physiological sensory reaction is enhanced for both perceptions [53].

**Figure 4:**
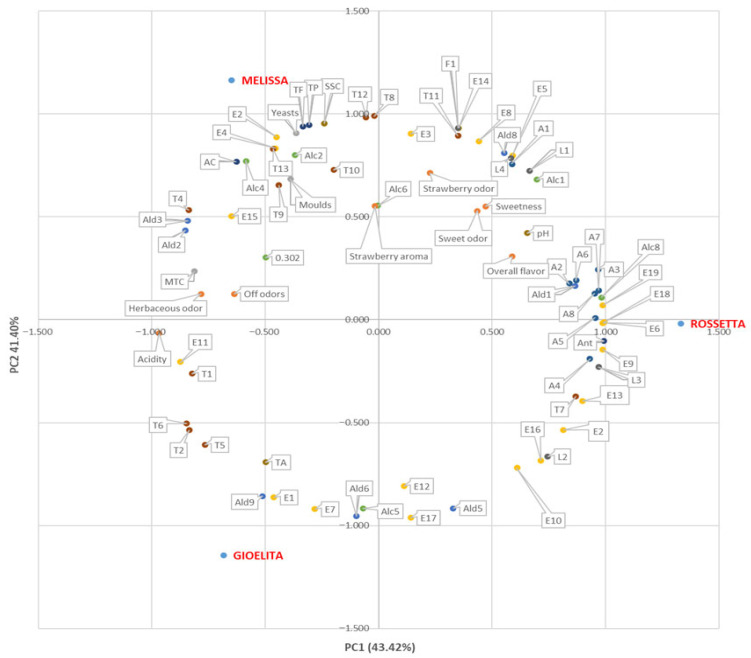
2D principal component analysis plot of the physico-chemical, qualitative, microbial and sensory attributes and volatile profiles in three strawberry cultivars (‘Rossetta’, ‘Melissa’ and ‘Gioelita’). Abbreviations are described in Table 1 and Table 2.

Figure 4 presents the 2D projection of scores and loading values, showing that the first two principal components explain 84.8% of the total variation in the dataset, with PC1 accounting for 43.4% and PC2 for 41.4%. In the 2D plot, the three strawberry cultivars are clearly separated. Specifically, ‘Rossetta’ is positioned with positive PC1 and slightly negative PC2 values, ‘Gioelita’ shows negative scores on both PC1 and PC2, and ‘Melissa’ displays negative PC1 and positive PC2 values (Figure 4).

‘Rossetta’ is mainly correlated with twelve esters, namely isopropyl butyrate (E3), butyl acetate (E5), isoamyl acetate (E6), butyl butyrate (E8), isopropyl hexanoate (E9), hexyl acetate (E10), methyl octanoate (E13), hexyl butyrate (E14), *trans*-2-hexenyl butyrate (E16), hexyl hexanoate (E18), octyl butyrate (E19), *trans*-2-hexenyl hexanoate (E20); with eight acids, namely propanoic acid (A1), 2-methyl propanoic acid (A2), butanoic acid (A3), 2-methyl-butanoic acid (A4), hexanoic acid (A5), heptanoic acid (A6), octanoic acid (A7), nonanoic acid (A8); with four lactones, namely γ-caprolactone (L1), γ-octalactone (L2), γ-decalactone (L3), γ-dodecalactone (L4); with two alcohols, namely 2-heptanol (Alc1) and benzenemethanol (Alc8); with hexanal (Ald1), myrtenal (T7), geraniol (T11), benzaldehyde (Ald8), mesifuran (F1); with the biochemical parameters anthocyanins (ANT) and pH; and with the sensory traits *Overall flavor*, *Sweet*, *Sweet odor*, and *Strawberry odor*.

Although this variety is directly associated with a high number of volatile acids (A1, A2, A3, A4–A8), the results of the panel test showed the lowest value for the sensory parameter *Acidity* and the highest value for both *Sweet* and *Sweet odor* (Figure 2), among others. These findings can be explained by the correlation with four lactones (L1–L4) and with the twelve esters (E3, E5, E6, E8-E10, E13, E14, E16, E18–E20). Lactones, in fact, are known to confer desirable “sweet,” “fruity,” or “peach-like” aroma to fruit [38]. Moreover, γ-decalactone (L3), particularly abundant in ‘Melissa’ with respect to the other varieties investigated (Table 2), is considered a strong contributor to the sweet and fruity flavor in strawberries [54], while γ-dodecalactone (L4) has been identified not only among the sweetness-enhancing compounds in strawberries, but also the volatile with the highest correlations with consumers’ liking [55]. Regarding the ester compounds, E6 and E18 have been observed only in ‘Rossetta’, while the five other components (E9, E13, E16, E19 and E20) were more abundant in these strawberries with respect to the other varieties (Table 2). With the exception of methyl octanoate (E13), described with citrus and tropical notes in some strawberry cultivars [43], the remaining esters are known to impart fruity and sweet notes to strawberries [56]. ‘Rossetta’ is also correlated with benzenemethanol (Alc8), which has been reported as a key odorant in strawberries, imparting floral notes [54]. Moreover, this variety is also correlated with mesifuran (F1), which, imparting a toffee-like and sugary flavor, is a key aroma compound in shaping the strawberry aroma [55].

Altogether, the outcomes of PCA can clarify why ‘Rossetta’ emerged as the most appreciated cultivar by panelists, with the highest score for *Overall flavor and Strawberry odor* (Figure 3). This result aligns with previous sensory evaluations that showed that a strawberry variety is highly valued if parameters, including sweet and aroma intensity, present high records [54].

Finally, the direct association of this cultivar with the biochemical parameter ANT agrees with the highest perceived color intensity by panelists (Figure 2) and the highest measured value of anthocyanin content (Table 1).

‘Gioelita’ is directly correlated with five esters, namely methyl butyrate (E1), methyl hexanoate (E7), 3-hexen-1-ol acetate (E11), trans-2-hexen-1-ol acetate (E12) and methyl-3-(methylthio) propanoate (E17); to three aldehydes, including nonanal (Ald5), 2-octenal (Ald6), and 2-nonenal (Ald9); to four terpenes, such as ß-myrcene (T1), D-limonene (T2), trans-linalool oxide (T5), and linalool L (T6); and to trans-2-hexen-1-ol (Alc5). Moreover, ‘Gioelita’ is also positively associated with the sensory trait *Acidity* and with the titratable acidity (TA).

Methyl butyrate (E1), methyl hexanoate (E7), and trans-2-hexen-1-ol acetate (E12) are highest in ‘Gioelita’ (Table 2). In particular, E1 and E7 have been previously described as among the main contributors to the overall appreciation of strawberry flavor [39,57]. On the other hand, linalool (T6) and *trans*-2-hexen-1-ol acetate (E12) impart a floral and citrus aroma to strawberries, while nonanal has been indicated to enhance the sweet sensation in this fruit [38,39,43,55]. Finally, ‘Gioelita’ appears correlated both to the TA parameter and to the sensory perception of *Acidity*, in line with the highest value of TA (Table 1) and with the sensory analysis results (Figure 2) obtained for this cultivar.

‘Melissa’ is positively associated with three esters, such as ethyl butyrate (E2), ethyl-3-methyl butyrate (E4), *cis*-3-hexenyl butyrate (E15); to six terpenes, including *cis*-linalool oxide (T4), *ß*-farnesene (T8), α-terpineol (T9), myrtenol (T10), nerolidol (T12), and eugenol (T13); to four alcohols, namely 1-hexanol (Alc2), *trans*-3-hexen-1-ol (Alc3), *cis*-3-hexen-1-ol (Alc4), and 6-methyl-5-hepten-2-ol (Alc6); and to aldehydes, including 2-pentenal (Ald2) and 2-hexenal (Ald3). ‘Melissa’ is also correlated to the sensory attributes of *Herbaceous odor, Strawberry aroma* and *Off odors*; to four quality parameters, namely total polyphenols (TP), total flavonoids (TF), antioxidant capacity (AC), and SSC (Brix); and to all the microbial traits, including TMC, yeasts and molds (Figure 4).

Specifically, all three esters associated with ‘Melissa’ strawberries were characterized by the same acyl-CoA group (butyrate group), and all are described to deliver fruity and sweet scents that resemble pineapple.

Sheng et al. [58], studying sixteen strawberry varieties from Jiangsu Province (China), classified the samples into four aroma types, namely, peachy, pineapple, fruity, and floral, according to the sensory descriptions of the principal VOCs associated with the variety [58]. Based on this study, ‘Melissa’ could be included in the pineapple-type group. Anyway, the highest *Herbaceous odor* sensation recorded by panelists (Figure 2) can be ascribed to the abundant levels of several green-leaf volatiles, including Alc2, Alc3, Alc4 and Ald3, observed in ‘Melissa’, which contribute to the herbal aroma in most strawberry varieties [39]. Notwithstanding, the correlation with the six terpenes (T4, T8-T10, T12, T13), which provide a floral aroma to strawberries, and with 2-pentenal (Ald2), reported to be strictly linked to consumers’ liking [55], gives this variety its overall balanced flavor, as emerged from the sensory evaluation (Figure 2).

Moreover, ‘Melissa’ is directly associated with the antioxidant capacity (AC), consistent with its positive correlation with the quality parameters related to total polyphenols (TP) and total flavonoids (TF), which are known to be biologically active metabolites [59].

Finally, ‘Melissa’ appears associated with TMC, yeasts and molds, in line with the microbiological quality evaluation (Figure 3). These data could explain the correlation of this cultivar with the *Off odors* that emerged in both panel test analysis and the PCA (Figure 2 and Figure 4).

## 4. Conclusions

The physico-chemical and qualitative traits, the volatile profiles, and the sensory attributes of three strawberry cultivars (‘Rossetta’, ‘Melissa’ and ‘Gioelita’) grown in soilless culture systems have been evaluated. Sixty-four volatile compounds, together with the physico-chemical, qualitative parameters and the sensory traits related to odor and taste perception, were submitted to PCA, which revealed a clear separation among the strawberry cultivars. Volatile compounds responsible for strawberry cultivar discrimination were identified. ‘Rossetta’ was associated with the highest number of VOCs, including twelve esters, four lactones and mesifuran, explaining its highest scores for *Overall flavor, Sweetness, Sweet odor*, and *Strawberry odor*. It also showed the highest *Color* score, which was consistent with its highest anthocyanin content. ‘Gioelita’ presented the highest amounts of methyl butyrate (E1) and methyl hexanoate (E7), key contributors to strawberry flavor, and it was correlated both to titratable acidity (TA) and to the sensory perception of *Acidity*. ‘Melissa’ was related to three butyrate esters (E2, E4 and E15), all described to deliver fruity and sweet scents that resemble pineapple; to several C6-volatiles (Alc2, Alc3, Alc4 and Ald3) responsible for herbaceous notes; to six terpenes (T4, T8–T10, T12, T13) conferring a floral aroma; and to Ald2, linked to consumer liking. These findings explain why this variety is characterized by an overall balanced flavor, as emerged from the sensory evaluation. Moreover, ‘Melissa’ was directly associated with the antioxidant capacity (AC), reflecting its higher levels of TP and TF compared to the other two cultivars, as well as the highest counts of TMC, yeasts, and molds. Overall, our findings offer valuable insights for future breeding programs focused not only on enhancing yield and general fruit quality but also on improving nutritional content and aroma characteristics.

Further studies regarding the cultivation of these three commercial strawberry cultivars by traditional methods are underway to clarify how the soilless cultivation employed in this study affected the quality and the sensory signatures of these products.

## Figures and Tables

**Table 1:** Physico-chemical and qualitative traits in ‘Rossetta’, ‘Melissa’ and ‘Gioelita’ strawberry cultivars. For each cultivar, samples were collected at two different harvest times (1 and 2). Values are expressed as mean ± standard deviation for the two harvests. Different letters within each line indicate statistically significant differences between cultivars, as determined by one-way ANOVA followed by Duncan post hoc test (*p* < 0.05).

Traits	‘Rossetta’	‘Melissa’	‘Gioelita’
Firmness (kg/cm^2^)	3.72 ± 0.11 ^a^	3.73 ± 0.28 ^a^	3.64 ± 0.21 ^a^
TSS (°Brix)	7.78 ± 0.03 ^b^	8.65 ± 0.15 ^c^	7.47 ± 0.06 ^a^
TA (mg CA/L)	4.37 ± 0.18 ^a^	4.41 ± 0.23 ^a^	4.97 ± 0.16 ^b^
pH	3.63 ± 0.06 ^b^	3.53 ± 0.15 ^ab^	3.40 ± 0.00 ^a^
L*	31.76 ± 1.73 ^a^	31.51 ± 1.05 ^a^	32.05 ± 0.72 ^a^
C	39.40 ± 3.45 ^a^	40.60 ± 3.15 ^a^	40.69 ± 1.04 ^a^
H°	26.12 ± 2.13 ^a^	25.16 ± 0.91 ^a^	27.22 ± 0.89 ^a^
TP (mg GAE/100 g FW)	155.63 ± 2.53 ^b^	222.95 ± 9.40 ^c^	138.28 ± 2.12 ^a^
TF (mg CE/100 g FW)	27.26 ± 0.46 ^b^	38.13 ± 0.28 ^c^	24.92 ± 0.25 ^a^
ANT (mg C3GE/100 g FW)	43.95 ± 1.02 ^a^	30.83 ± 0.04 ^c^	32.09 ± 0.14 ^b^
AC (µmol TE/g FW)	9.73 ± 0.06 ^a^	13.83 ± 0.60 ^c^	10.50 ± 0.18 ^b^

**Table 2:** Volatile compounds detected in the three strawberry cultivars, ‘Rossetta’ (R), ‘Melissa’ (M), and ‘Gioelita’ (G), grown in a soilless system. For each cultivar, samples were collected at two different harvest times (1 and 2). Values are expressed as mean ± standard deviation of 3-octanol equivalents, µg kg^−1^.

Volatile Compounds	Code	^a^ RIsp/ ^b^ LRIt	^c^ ID	R1	R2	M1	M2	G1	G2	*p*
**Esters**										
Methyl butyrate	E1	989/989	LRI/MS/S	33.6 ± 0.5 ^a^	33.7 ± 1.2 ^a^	33.9 ± 2.4 ^a^	33.7 ± 2.4 ^a^	62.6 ± 3.5 ^b^	64.9 ± 2.4 ^c^	***
Ethyl butyrate	E2	1037/1037	LRI/MS/S	2.0 ± 0.2 ^a^	2.4 ± 0.2 ^c^	6.9 ± 0.7 ^d^	7.1 ± 0.7 ^bc^	2.5 ± 0.2 ^abc^	2.3 ± 0.3 ^ab^	***
Isopropyl butyrate	E3	1046/1044	LRI/MS/S	3.6 ± 0.2 ^a^	3.6 ± 0.2 ^a^	4.9 ± 0.1 ^b^	5.0 ± 0.1 ^b^	3.5 ± 1.3 ^a^	3.1 ± 0.5 ^a^	***
Ethyl 3-methylbutyrate	E4	1073/1072	LRI/MS/S	ND	ND	0.2 ± 0.0 ^a^	0.2 ± 0.0 ^a^	ND	ND	***
Butyl acetate	E5	1074/1074	LRI/MS/S	0.7 ± 0.1 ^a^	0.8 ± 0.1 ^b^	0.7 ± 0.1 ^a^	0.7 ± 0.1 ^a^	ND	ND	***
Isoamyl acetate	E6	1114/1114	LRI/MS/S	0.4 ± 0.1 ^a^	0.4 ± 0.1 ^a^	ND	ND	ND	ND	***
Methyl hexanoate	E7	1190/1190	LRI/MS/S	35.8 ± 1.2 ^b^	33.7 ± 1.2 ^ab^	29.2 ± 2.0 ^a^	29.0 ± 2.1 ^a^	66.2 ± 3.1 ^c^	65.1 ± 3.6 ^c^	***
Butyl butyrate	E8	1232/1232	LRI/MS/S	2.5 ± 0.5 ^b^	2.7 ± 0.1 ^c^	2.5 ± 0.1 ^b^	2.6 ± 0.1 ^b^	0.6 ± 0.1 ^a^	0.6 ± 0.2 ^b^	***
Isopropyl hexanoate	E9	1284/1284	LRI/MS/S	2.6 ± 0.5 ^b^	2.6 ± 0.1 ^b^	ND	ND	0.4 ± 0.1 ^a^	0.4 ± 0.1 ^a^	***
Hexyl acetate	E10	1269/1269	LRI/MS/S	4.1 ± 1.8 ^d^	4.6 ± 0.1 ^ab^	2.4 ± 0.1 ^a^	2.4 ± 0.1 ^a^	5.3 ± 1.4 ^c^	5.0 ± 0.7 ^bc^	***
3-Hexen-1-ol acetate	E11	1326/1322	LRI/MS/S	0.4 ± 0.4 ^ab^	0.5 ± 0.1 ^a^	1.0 ± 0.0 ^bc^	0.8 ± 0.1 ^ab^	1.0 ± 0.1 ^c^	1.0 ± 0.3 ^bc^	***
2-Hexen-1-ol acetate	E12	1344/1344	LRI/MS/S	11.8 ± 1.5 ^b^	11.3 ± 1.4 ^b^	7.4 ± 0.1 ^a^	7.1 ± 0.1 ^a^	20.1 ± 6.2 ^c^	18.7 ± 6.3 ^ab^	**
Methyl octanoate	E13	1396/1395	LRI/MS/S	0.7 ± 0.2 ^b^	0.8 ± 0.1 ^b^	ND	ND	0.2 ± 0.1 ^a^	0.3 ± 0.1 ^a^	***
Hexyl butyrate	E14	1426/1426	LRI/MS/S	7.0 ± 1.1 ^b^	8.1 ± 1.4 ^d^	9.1 ± 0.1 ^e^	9.3 ± 0.1 ^e^	1.3 ± 0.4 ^b^	1.0 ± 0.1 ^a^	***
*cis*-3-Hexenyl butyrate	E15	1431/1431	LRI/MS/S	0.4 ± 0.1 ^a^	0.4 ± 0.0 ^a^	0.6 ± 0.1 ^b^	0.7 ± 0.1 ^b^	0.5 ± 0.1 ^b^	0.5 ± 0.1 ^b^	***
*trans*-2-Hexenyl butyrate	E16	1478/1477	LRI/MS/S	8.6 ± 0.8 ^c^	9.6 ± 0.6 ^c^	3.2 ± 0.4 ^a^	3.4 ± 0.4 ^a^	7.9 ± 0.2 ^b^	7.4 ± 0.5 ^b^	***
Methyl-3-(methylthio) propanoate	E17	1525/1525	LRI/MS	0.5 ± 0.1 ^bcd^	0.5 ± 0.1 ^bc^	0.2 ± 0.1 ^ab^	0.1 ± 0.0 ^a^	0.6 ± 0.1 ^d^	0.5 ± 0.1 ^bc^	***
Hexyl hexanoate	E18	1608/1607	LRI/MS/S	1.0 ± 0.0 ^a^	1.0 ± 0.1 ^a^	ND	ND	ND	ND	***
Octyl butyrate	E19	1624/1622	LRI/MS/S	1.8 ± 0.1 ^c^	2.1 ± 0.1 ^c^	0.4 ± 0.1 ^b^	0.4 ± 0.1 ^b^	0.2 ± 0.1 ^a^	0.2 ± 0.0 ^a^	***
*trans-*2-Hexenyl hexanoate	E20	1669/1670	LRI/MS/S	0.9 ± 0.2 ^c^	0.8 ± 0.1 ^c^	0.4 ± 0.1 ^a^	0.4 ± 0.0 ^a^	0.5 ± 0.1 ^b^	0.7 ± 0.1 ^b^	***
Benzyl acetate	E21	1731/1731	LRI/MS/S	1.5 ± 0.3 ^a^	1.6 ± 0.1 ^a^	1.3 ± 0.6 ^a^	1.8 ± 0.6 ^a^	1.3 ± 0.1 ^a^	1.4 ± 0.1 ^a^	ns
**Aldehydes**										
Hexanal	Ald1	1084/1084	LRI/MS/S	20.4 ± 0.7 ^a^	21.9 ± 1.1 ^b^	16.8 ± 0.4 ^ab^	16.5 ± 0.4 ^b^	16.9 ± 1.4 ^ab^	14.2 ± 1.6 ^ab^	***
*trans*-2-Pentenal	Ald2	1085/1085	LRI/MS/S	0.7 ± 0.5 ^bc^	0.8 ± 0.3 ^a^	1.9 ± 0.1 ^c^	1.7 ± 0.1 ^c^	1.4 ± 0.2 ^bc^	1.1 ± 0.2 ^b^	***
2-Hexenal	Ald3	1242/1240	LRI/MS/S	191.6 ± 3.6 ^a^	206.7 ± 7.1 ^b^	309.9 ± 8.1 ^bc^	302.8 ± 8.0 ^c^	263.8 ± 5.6 ^b^	244.3 ± 5.9 ^b^	***
2-Heptenal	Ald4	1341/1342	LRI/MS/S	1.1 ± 0.4 ^a^	0.8 ± 0.1 ^a^	0.6 ± 0.1 ^a^	0.6 ± 0.1 ^a^	0.7 ± 0.1 ^a^	0.8 ± 0.1 ^a^	ns
Nonanal	Ald5	1404/1404	LRI/MS/S	0.5 ± 0.0 ^a^	0.6 ± 0.1 ^a^	ND	ND	0.6 ± 0.3 ^b^	0.8 ± 0.1 ^c^	***
2-Octenal	Ald6	1455/1455	LRI/MS/S	1.0 ± 0.1 ^c^	1.1 ± 0.1 ^ab^	0.9 ± 0.1 ^a^	0.7 ± 0.0 ^b^	1.3 ± 0.1 ^d^	1.3 ± 0.1 ^d^	***
Decanal	Ald7	1510/1511	LRI/MS/S	0.9 ± 0.1 ^a^	0.8 ± 0.1 ^b^	0.8 ± 0.1 ^b^	0.7 ± 0.1 ^ab^	0.9 ± 0.1 ^a^	0.8 ± 0.1 ^b^	ns
Benzaldehyde	Ald8	1520/1521	LRI/MS/S	1.1 ± 0.2 ^b^	1.0 ± 0.1 ^b^	1.1 ± 0.1 ^b^	1.1 ± 0.1 ^b^	0.5 ± 0.1 ^a^	0.5 ± 0.1 ^a^	***
2-Nonenal	Ald9	1531/1531	LRI/MS/S	ND	ND	ND	ND	0.4 ± 0.1 ^b^	0.3 ± 0.1 ^a^	***
**Alcohols**										
2-Heptanol	Alc1	1326/1325	LRI/MS/S	0.4 ± 0.2 ^b^	0.4 ± 0.1 ^b^	0.3 ± 0.1 ^a^	0.3 ± 0.0 ^a^	ND	ND	***
1-Hexanol	Alc2	1365/1365	LRI/MS/S	10.1 ± 2.1 ^c^	9.6 ± 1.3 ^ab^	13.0 ± 0.1 ^a^	13.6 ± 0.1 ^d^	9.9 ± 1.5 ^bc^	9.7 ± 1.3 ^ab^	***
*trans*-3-Hexen-1-ol	Alc3	1398/1398	LRI/MS/S	0.3 ± 0.1 ^d^	0.2 ± 0.1 ^a^	0.4 ± 0.1 ^cd^	0.4 ± 0.0 ^bc^	0.3 ± 0.1 ^cb^	0.3 ± 0.0 ^ab^	***
*cis*-3-Hexen-1-ol	Alc4	1400/1401	LRI/MS/S	0.5 ± 0.1 ^a^	0.5 ± 0.1 ^a^	1.7 ± 0.1 ^b^	2.2 ± 0.1 ^c^	0.7 ± 0.1 ^a^	0.9 ± 0.2 ^b^	***
*trans*-2-Hexen-1-ol	Alc5	1416/1415	LRI/MS/S	20.5 ± 1.9 ^a^	21.8 ± 1.8 ^b^	18.8 ± 1.8 ^a^	20.2 ± 0.3 ^a^	26.0 ± 0.8 ^bc^	25.1 ± 1.4 ^b^	***
6-Methyl-5-hepten-2-ol	Alc6	1464/1464	LRI/MS/S	0.4 ± 0.1 ^a^	0.4 ± 0.1 ^a^	0.4 ± 0.1 ^a^	0.5 ± 0.1 ^b^	0.4 ± 0.1 ^a^	0.4 ± 0.0 ^a^	***
1-Octanol	Alc7	1561/1561	LRI/MS/S	0.5 ± 0.0 ^a^	0.6 ± 0.1 ^b^	0.5 ± 0.1 ^ab^	0.5 ± 0.1 ^ab^	0.5 ± 0.1 ^ab^	0.4 ± 0.1 ^a^	ns
Benzenemethanol	Alc8	1864/1865	LRI/MS/S	1.6 ± 0.2 ^c^	1.7 ± 0.1 ^c^	1.0 ± 0.3 ^b^	1.1 ± 0.1 ^b^	0.8 ± 0.1 ^a^	0.8 ± 0.2 ^a^	***
**Acids**										
Propanoic acid	A1	1534/1534	LRI/MS/S	0.8 ± 0.3 ^bc^	0.8 ± 0.1 ^bc^	0.7 ± 0.1 ^c^	0.7 ± 0.1 ^c^	0.2 ± 0.0 ^a^	0.1 ± 0.1 ^a^	***
2-Methylpropanoic acid	A2	1544/1540	LRI/MS/S	1.1 ± 0.1 ^bc^	1.4 ± 0.1 ^d^	1.0 ± 0.2 ^c^	1.1 ± 0.2 ^bc^	1.1 ± 0.6 ^a^	1.0 ± 0.3 ^b^	***
Butanoic acid	A3	1631/1631	LRI/MS/S	8.3 ± 0.1 ^cd^	8.8 ± 0.1 ^d^	3.4 ± 1.2 ^c^	3.5 ± 1.1 ^c^	1.7 ± 0.1 ^b^	1.4 ± 0.4 ^a^	***
2-Methylbutanoic acid	A4	1701/1700	LRI/MS/S	16.4 ± 0.8 ^b^	16.4 ± 0.5 ^b^	12.1 ± 0.5 ^a^	12.0 ± 0.5 ^a^	13.5 ± 1.2 ^c^	12.5 ± 1.5 ^bc^	***
Hexanoic acid	A5	1848/1848	LRI/MS/S	122.6 ± 10 ^ab^	154.4 ± 12 ^b^	86.3 ± 8.2 ^a^	85.6 ± 8.0 ^a^	76.5 ± 4.2 ^c^	84.7 ± 4.4 ^c^	***
Heptanoic acid	A6	1952/1952	LRI/MS/S	0.4 ± 0.1 ^cd^	0.5 ± 0.1 ^d^	0.2 ± 0.1 ^b^	0.2 ± 0.1 ^ab^	0.1 ± 0.0 ^a^	0.1 ± 0.0 ^a^	***
Octanoic acid	A7	2073/2074	LRI/MS/S	1.6 ± 0.6 ^b^	1.9 ± 0.1 ^c^	0.5 ± 0.1 ^ab^	0.6 ± 0.1 ^ab^	0.2 ± 0.1 ^a^	0.4 ± 0.1 ^ab^	***
Nonanoic acid	A8	2174/2174	LRI/MS/S	0.7 ± 0.1 ^a^	1.1 ± 0.2 ^b^	0.2 ± 0.1 ^a^	0.2 ± 0.1 ^a^	0.1 ± 0.0 ^a^	0.1 ± 0.0 ^a^	***
**Terpens**										
ß-Myrcene	T1	1196/1196	LRI/MS/S	0.6 ± 0.1 ^a^	0.6 ± 0.1 ^a^	2.0 ± 0.3 ^c^	2.0 ± 0.4 ^c^	1.4 ± 0.1 ^b^	1.3 ± 0.1 ^b^	***
D-Limonene	T2	1199/1200	LRI/MS/S	2.5 ± 0.3 ^a^	2.8 ± 0.1 ^b^	4.4 ± 0.5 ^c^	5.8 ± 0.6 ^c^	9.7 ± 2.2 ^d^	11.2 ± 2.3 ^d^	***
6-Methyl-5-hepten-2-one	T3	1338/1338	LRI/MS/S	24.3 ± 0.2 ^a^	24.4 ± 0.8 ^a^	40.4 ± 2.9 ^b^	40.9 ± 3.0 ^b^	25.8 ± 2.8 ^c^	25.5 ± 2.6 ^c^	***
*cis*-Linalool oxide	T4	1467/1467	LRI/MS/S	0.7 ± 0.1 ^c^	0.7 ± 0.1 ^c^	0.3 ± 0.1 ^a^	0.2 ± 0.1 ^a^	0.5 ± 0.1 ^b^	0.5 ± 0.1 ^b^	***
*trans*-Linalool oxide	T5	1471/1471	LRI/MS/S	0.4 ± 0.1 ^b^	0.2 ± 0.1 ^a^	0.7 ± 0.1 ^c^	0.7 ± 0.1 ^c^	ND	ND	***
Linalool L	T6	1553/1557	LRI/MS/S	4.7 ± 0.5 ^ba^	4.3 ± 0.3 ^a^	6.2 ± 1.2 ^c^	6.5 ± 1.2 ^c^	5.1 ± 0.6 ^b^	5.6 ± 0.7 ^b^	***
Myrtenal	T7	1634/1633	LRI/MS/S	0.2 ± 0.0 ^a^	0.3 ± 0.1 ^a^	1.7 ± 1.2 ^c^	0.6 ± 1.2 ^b^	0.1 ± 0.0 ^a^	0.1 ± 0.0 ^a^	***
ß-Farnesene	T8	1699/1698	LRI/MS/S	0.4 ± 0.1 ^a^	0.4 ± 0.1 ^a^	0.4 ± 0.1 ^a^	0.4 ± 0.1 ^a^	0.2 ± 0.0 ^b^	0.2 ± 0.0 ^b^	***
α-Terpineol	T9	1700/1700	LRI/MS/S	10.7 ± 0.5 ^b^	9.1 ± 0.6 ^b^	14.3 ± 1.0 ^c^	16.2 ± 1.2 ^c^	4.4 ± 1.3 ^a^	5.9 ± 1.6 ^a^	***
Myrtenol	T10	1790/1790	LRI/MS/S	ND	ND	0.1 ± 0.1 ^a^	0.2 ± 0.1 ^b^	ND	ND	***
Geraniol	T11	2045/2045	LRI/MS/S	0.6 ± 0.1 ^a^	0.6 ± 0.1 ^a^	2.0 ± 0.3 ^c^	2.0 ± 0.4 ^c^	1.4 ± 0.1 ^b^	1.3 ± 0.1 ^b^	***
Nerolidol	T12	2055/2055	LRI/MS/S	10.7 ± 0.3 ^a^	9.1 ± 0.6 ^b^	14.3 ± 0.5 ^c^	14.6 ± 0.6 ^c^	4.4 ± 0.2 ^d^	4.2 ± 0.1 ^d^	***
Eugenol	T13	2167/2167	LRI/MS/S	34.9 ± 0.2 ^a^	36.4 ± 2.8 ^a^	51.4 ± 2.9 ^b^	51.9 ± 3.0 ^b^	68.8 ± 8.8 ^c^	74.5 ± 8.6 ^c^	***
**Lactones**										
γ-Caprolactone	L1	1717/1711	LRI/MS/S	0.7 ± 0.1 ^b^	0.7 ± 0.1 ^b^	0.6 ± 0.1 ^ab^	0.7 ± 0.1 ^b^	0.4 ± 0.1 ^a^	0.4 ± 0.1 ^a^	***
γ-Octalactone	L2	1967/1965	LRI/MS/S	0.4 ± 0.4 ^b^	0.4 ± 0.7 ^b^	ND	ND	0.2 ± 0.0 ^a^	0.2 ± 0.0 ^a^	***
γ-Decalactone	L3	2195/2195	LRI/MS/S	32.3 ± 5.2 ^c^	35.3 ± 5.3 ^c^	0.3 ± 0.1 ^a^	0.3 ± 0.1 ^a^	8.0 ± 1.1 ^ab^	8.3 ± 1.2 ^b^	***
γ-Dodecalactone	L4	2382/2388	LRI/MS/S	1.4 ± 0.4 ^c^	1.2 ± 2.7 ^c^	1.1 ± 0.4 ^b^	1.3 ± 0.6 ^ab^	0.6 ± 0.3 ^a^	0.6 ± 0.3 ^a^	***
**Furanones**										***
Mesifuran	F1	1603/1603	LRI/MS	50.0 ± 4.2 ^c^	56.2 ± 3.7 ^d^	66.3 ± 4.2 ^bc^	65.2 ± 4.2 ^bc^	6.8 ± 1.6 ^a^	6.9 ± 1.8 ^ab^	***

^a^ LRIsp: linear retention index relative n-alkanes (C_8_ to C_20_) on a HP-Innowax capillary column; ^b^ LRIt: linear retention index values reported in the literature for equivalent capillary column; ^c^ ID: identification methods, including linear retention index, LRI; mass spectrum, MS; commercial standard (S). Different letters within each line indicate statistically significant differences between groups (*p* < 0.05). Volatiles indicated by ** and *** have significant *p*-values (** 0.01 and *** 0.001) by Tukey’s post hoc test; ns: not significant; ND: not detected.

## Data Availability

The data used to support the findings of this study can be made available by the corresponding author upon request.

## Supplementary Materials

The following supporting information can be downloaded at: https://www.mdpi.com/article/10.3390/foods15061072/s1, Table S1: Sensory attributes of strawberry cvs ‘Melissa’, ‘Gioelita’ and ‘Rossetta’ reported on the evaluation sheet, their definitions and references. The number in brackets indicates the intensity scale value. Table S2: Sensory attributes (mean values ± sd) of strawberry cvs ‘Melissa’, ‘Gioelita’ and ‘Rossetta’. Different letters within each line indicate statistically significant differences between groups (*p* < 0.05), as determined by one-way ANOVA followed by Tukey’s HSD post hoc test (*** *p* < 0.001; ** *p* < 0.01; * *p*< 0.05). Table S3: Evaluation of panel performance as reported by 3-way ANOVA performed with Panel Check. The interaction Assessor x Product is significant only for the “Acidity” attribute. This interaction measures the consistency of individual assessors across different products. It indicates whether panelists are using the rating scales differently for specific products. If the *p*-value is not significant it means that the panel is in consensus: panel members perceive the product differences similarly. The interaction of Product x Replicate is not significant for all the attributes. This interaction measures whether the assessors’ evaluation of a specific product changed from the first time they tasted/tested it to a subsequent session (replication). A not significant *p*-value suggests that the panel evaluates the products the same way across all replicates. The interaction Assessor x Replicate has a significant *p*-value only for the ‘Herbaceous odor’ and ‘Acidity’ attributes. This means that the panel for those attributes does not have the same grade mean for all the products.

## Abbreviations

The following abbreviations are used in this manuscript:

VOCs	Volatile organic compounds
HS-SPME	Headspace solid-phase microextraction
GC-MS	Gas chromatography-mass spectrometry
TSS	Total soluble solids
TA	Titratable acidity
TP	Total polyphenols
TF	Total flavonoids
AC	Antioxidant capacity
ANT	Anthocyanins
TMC	Total mesophilic count
