# Supplementary material for: Strawberry Production in Soilless Culture Systems: A Comparative Analysis of Volatile Metabolites, Quality, and Sensory Traits in Three Cultivars
Source: Foods. 2026 Mar 18;15(6):1072. doi: 10.3390/foods15061072 (PMC13025174; doi:10.3390/foods15061072)
Supplement: Supplementary file 1 [file foods-15-01072-s001.zip › foods-4188034-supplementary.pdf]

Article

Strawberry production in soilless culture systems: volatile metabolites, quality and sensory traits

Livia Malorni[1], Tiziana Di Renzo[1], Cristina Matarazzo[2], Milena Petriccione[3], Elvira Ferrara[3], Giuseppe Capriolo[3], Gianluca Baruzzi[4], Paolo Sbrighi[4], Rosaria Cozzolino[1*]

[1] Institute of Food Science, National Council of Research, ISA-CNR Via Roma 64, Avellino; livia.malorni@isa.cnr.it (L.M.); tiziana.direnzo@isa.cnr.it (T.D.R.); rosaria.cozzolin@isa.cnr.it (R.C.)
[2] Department of Agriculture, Environmental and Food Sciences, University of Molise, Campobasso, Italy; c.matarazzo3@studenti.unimol.it (C.M.);
[3] CREA - Research Centre for Olive, Fruit and Citrus Crops Via Torrino 2, 81100 Caserta, Italy; milena.petriccione@crea.gov.it (M.P.); elivira.ferrara@crea.gov.it (E.F.); giuseppe.capriolo@crea.gov.it (G.C.)
[4] CREA - Research Centre for Olive, Fruit and Citrus Crops Via La Canapona, 1 bis, 47121, Forlì, Italy gianluca.barbuzi@crea.gov.it (G.B.); paolo.sbrighi@crea.gov.it (P.S.)
* Correspondence: rosaria.cozzolino@isa.cnr.it (R.C.); Tel.: +39-0825299111

SUPLLEMENTARY MATERIALS

Table S1: Sensory attributes of strawberry cvs 'Melissa', 'Gioelita' and 'Rossetta' reported on the evaluation sheet, their definitions and references. The number in brackets indicates the intensity scale value.

Sensory Attributes	Definition	References
**Appearance**		
Colour	Degree of the red colour	White (1); Red (9)
Visual freshness	Evaluating homogeneity of colour on fruit (presence/absence of dark spot)	Non-uniform (1); Uniform (9)
Surface Glossiness	Evaluating intensity of the light reflected from fruit surface	Dull (1); Bright (9)
Seed size	Apparent size of the seeds	Small (1); Big (9)
Calyx Freshness	Degree of perceived freshness of the calyx	Brown/Old (1); Green/Fresh (9)
**Aroma**		
Sweet odor	Measuring the aroma associated with any fruit	Distilled water (1); 1 g vanillin in 500 ml distilled water (9)
Herbaceous odor	Measuring intensity of characteristic odour of fresh cut green grass	Distilled water (1); 25 g of fresh parsley rinsed, chopped, and added to 300 mL water; liquid part filtered after 15 min (9)
Strawberry odor	Evaluating intensity of characteristic odour of ripe fruit	Distilled water (1); Strawberry purée made with a variety of ripe fruit crushed before each panel (9)
Off odors	Intensity of strawberry fermentation odour and other off odors (mould; pungent)	Distilled water (1); Strawberry purée made with a variety of ripe fruit crushed and left overnight at 25°C (9)
**Texture – mouth feel**		
Juiciness	Measuring the amount of liquid released during chewing.	Banana (1); cucumber (5); watermelon (9)
Fruit Crunchiness	Evaluating the resistance degree of the pulp structure during chewing	Banana (1); apple (5); carrot (9)
Seed perception	The degree of seed perception	None (1); Noticeable (9)
Sweetness	Measuring intensity of the sugar specific sensation	1.0% sucrose solution (1)<br>5.0% sucrose solution (9)
Acidity	Measuring intensity of the sensation caused by acidic substances	0.05% citric acid solution (1)<br>0.08% citric acid solution (9)
Strawberry aroma	Evaluating intensity of typical fruit aroma retro-nasally perceived	Distilled water (1); Strawberry purée made with a variety of ripe fruit crushed before each panel (9)

Overall flavor	Measuring intensity of retro-nasally perceived aromas, 1 minute after swallowing	None (1); Strong (9)

Table S2: Sensory attributes (mean values ± sd) of strawberry cvs 'Melissa', Gioelita' and 'Rossetta'. Different letters within each line indicate statistically significant differences between groups ($p < 0,05$), as determined by one-way ANOVA followed by Tukey's HSD post hoc test (***$p<0,001$; **$p<0,01$; *$p< 0,05$)

Sensory attributes	Rossetta	Melissa	Gioelita	*p*
Colour	6,5 ± 0.4^{c}	4,7 ± 0,1^{a}	5,5 ± 0,3^{b}	***
Visual freshness	7,4 ± 0.1^{b}	7,0 ± 0.2^{ab}	6,8 ± 0,3^{a}	**
Surface Glossiness	6,4 ± 0.3^{a}	5,7 ± 0,3^{a}	6,2 ± 0.3^{a}	*
Seed size	5,4± 0.2^{a}	6,5 ± 0.1^{b}	5,5 ± 0.0^{a}	***
Calyx Freshness	7,5 ± 0,1^{a}	7,2 ± 0,2^{a}	7,0 ± 0.4^{a}	ns
Sweet odor	6,1 ± 0,3^{a}	6,0 ± 0,2^{a}	5,6 ± 0,4^{a}	ns
Herbaceous odor	4,6 ± 0,0^{a}	4,9 ± 0,1^{a}	4,8 ± 0,2^{a}	ns
Strawberry odor	6,8 ± 0,2^{a}	6,9 ± 0,2^{a}	6,6 ± 0.1^{a}	ns
Off odors	1,0 ± 0,0^{a}	1,2 ± 0,0^{a}	1,2 ± 0,2^{a}	ns
Juiciness	6,3 ± 0,1^{a}	6,1 ± 0,3^{a}	6,3 ± 0,4^{a}	ns
Fruit Crunchiness	5,5 ± 0,2^{a}	5,4 ± 0,3^{a}	5,8 ± 0,1^{a}	ns
Seed perception	5,3 ± 0,1^{a}	5,9 ± 0,3^{a}	5,9 ± 0,4^{a}	ns
Sweetness	5,6 ± 0,3^{a}	5,5 ± 0,3^{a}	5,0 ± 0,3^{a}	ns
Acidity	4,6 ± 0,2^{a}	5,6 ± 0,1^{b}	5,7 ± 0,1^{b}	***
Strawberry aroma	6,4 ± 0.3^{a}	6,5 ± 0.1^{a}	6,3 ± 0,1^{a}	ns
Overall flavor	6,5 ± 0.1^{a}	6,4 ± 0.1^{a}	6,3 ± 0,1^{a}	ns

Table S3. Evaluation of Panel Performance as reported by 3-way ANOVA performed with Panel Check.

The interaction **Assessor x Product** is significant only for the "Acidity" attribute. This interaction measures the consistency of individual assessors across different products. It indicates whether panelists are using the rating scales differently for specific products. If the p value is not significant it means that the panel is in consensus: panel members perceive the product differences similarly.

The interaction **Product x Replicate** is not significant for all the attributes. This interaction measures whether the assessors' evaluation of a specific product changed from the first time they tasted/tested it to a subsequent session (replication). A not significant p-value suggests that the panel evaluates the products the same way across all replicates.

The interaction **Assessor x Replicate** has a significant p-value only for the 'Herbaceous odor' and 'Acidity' attribute. This means that the panel, for those attributes, does not have the same grade mean for all the products.

	Color	Surface Gloss.	Seeds Size	Visual Fresh.	Calyx Fresh.	Sweet odor	Herb. odor	Straw. odor	Off odors	Fruit Crunch.	Juici.	Seed perc.	Sweet.	Acidity	Straw. aroma	Overall flavor
**Melissa**	4,729	5,688	6,479	6,958	7,229	6,021	4,875	6,917	1,229	5,396	6,104	5,896	5,479	5,583	6,542	6,375
**Rossetta**	6,521	6,437	5,438	7,375	7,458	6,104	4,583	6,833	1,021	5,479	6,292	5,271	5,583	4,563	6,438	6,479
**Gioelita**	5,542	6,167	5,521	6,792	6,979	5,583	4,833	6,563	1,187	5,771	6,271	5,854	4,979	5,688	6,333	6,292
**Assessor Effect (F value)**	3,89	3,49	4,39	8,45	2,38	6,75	6,19	1,78	3,07	7,1	6,53	3,88	4,18	8,05	3,18	3,83
**p-values**	0,001	0,004	0,001	0,001	0,029	0,003	0	0,088	0,004	0	0	0,002	0,002	0	0,014	0,001
**Product Effect (F value)**	15,67	2,78	13,47	5,52	1,89	3,19	0,89	0,9	2,23	2,11	0,38	3,17	1,81	10,88	0,41	0,3
**p-values**	0,008	0,153	0	0,009	0,222	0,056	0,421	0,419	0,236	0,139	0,724	0,056	0,235	0	0,665	0,746
**Replicate Effect (F value)**	0,24	0,01	0,62	1,28	0,42	1,45	0,33	0,04	2,31	0,28	0,04	4,18	0,54	0,16	2,36	0,51
**p-values**	0,789	0,989	0,575	0,538	0,675	0,424	0,735	0,957	0,229	0,828	0,963	0,09	0,609	0,853	0,233	1
**Assessor*Product Interaction (F value)**	1,22	1,36	1,55	0,73	1,55	0,77	1,51	1,3	1	0,56	0,68	1,46	1,52	2,06	1,25	1,01
**p-values**	0,255	0,157	0,074	0,824	0,075	0,781	0,089	0,192	0,482	0,957	0,878	0,105	0,084	0,009	0,229	0,468
**Product*Replicate Interaction (F value)**	1,50	2,27	0,18	0,62	1,11	0,85	0,14	0,47	1,19	0,73	1,29	0,23	1,05	0,48	0,29	0,16
**p-values**	0,214	0,072	0,95	0,65	0,358	0,501	0,968	0,757	0,326	0,574	0,283	0,921	0,387	0,754	0,884	0,958

**Assessor*Replicate Interaction (F value)**	0,99	1,4	1,39	1,12	1,39	1,04	2,72	0,86	0,47	0,72	0,62	1,36	1,06	1,83	1,03	0,72
**p-values**	0,497	0,136	0,137	0,344	0,14	0,441	0	0,671	0,986	0,84	0,924	0,154	0,411	0,024	0,444	0,841
**LSD**	0,84	0,83	0,46	0,37	0,59	0,45	0,48	0,57	0,31	0,39	0,98	0,57	0,81	0,54	0,47	0,5
**Bonferroni LSD**	1,170	1,13	0,57	0,46	0,78	0,56	0,6	0,7	0,45	0,49	1,7	0,7	1,08	0,68	0,58	0,62
